# Clathrin complexes with the inhibitor kappa B kinase signalosome: imaging the interactome

**DOI:** 10.14814/phy2.12035

**Published:** 2014-07-03

**Authors:** Fabia Gamboni, Guillermo A. Escobar, Ernest E. Moore, Monika Dzieciatkowska, Kirk C. Hansen, Sanchayita Mitra, Trevor A. Nydam, Christopher C. Silliman, Anirban Banerjee

**Affiliations:** 1 Department of Surgery, University of Colorado Denver, Aurora, Colorado; 2 Department of Surgery, University of Arkansas for Medical Sciences, Little Rock, Arkansas; 3 Department of Surgery, Denver Health Medical Center, Denver, Colorado; 4 UC Denver Mass Spectrometry and Proteomics Facility, Aurora, Colorado; 5 Department of Biochemistry and Molecular Genetics, UC Denver Mass Spectrometry and Proteomics Facility, Aurora, Colorado; 6 Belle Bonfils Blood Center, Denver, Colorado

**Keywords:** Affinity proteomics, fluorescent resonance energy transfer, Hsp90, NEMO

## Abstract

Many receptors involved with innate immunity activate the inhibitor kappa B kinase signalosome (IKK). The active complex appears to be assembled from the two kinase units, IKK*α* and IKK*β* with the regulatory protein NEMO. Because we previously found that RNA silencing of clathrin heavy chains (CHC), in transformed human lung pneumocytes (A549), decreased TNF*α*‐induced signaling and phosphorylation of inhibitor kappa B (I*κ*B), we hypothesized that CHC forms cytoplasmic complexes with members of the IKK signalosome. Widely available antibodies were used to immunoprecipitate IKK*α* and NEMO interactomes. Analysis of the affinity interactomes by mass spectrometry detected clathrin with both baits with high confidence. Using the same antibodies for indirect digital immunofluorescence microscopy and FRET, the CHC–IKK complexes were visualized together with NEMO or HSP90. The natural variability of protein amounts in unsynchronized A549 cells was used to obtain statistical correlation for several complexes, at natural levels and without invasive labeling. Analyses of voxel numbers indicated that: (i) CHC–IKK complexes are not part of the IKK signalosome itself but, likely, precursors of IKK–NEMO complexes. (ii) CHC–IKK*β* complexes may arise from IKK*β*–HSP90 complexes.

## Abstract (precis)

Clathrin forms complexes with IKKa, IKKb, and NEMO, but apparently not the canonical signalosome. These complexes are identified, for the first time, by affinity proteomics and triple FRET without altering molecular structure.

## Introduction

Phosphorylation and subsequent degradation of the inhibitor kappa B (I*κ*B) protein by its upstream kinase (I*κ*B kinase; IKK) are a key step for a wide variety of pathways regulating inflammation, cancer, or cell survival (Greten et al. 2004; Karin and Greten 2005; Viatour et al. 2005; Scheidereit 2006; Hayden and Ghosh 2008). The IKK signalosome conducts signaling from several receptors including those activated by lipopolysaccharide, TNF*α* IL 1*β*, and growth factors (DiDonato et al. 1997; Dinarello 2000; Karin and Ben‐Neriah 2000; Chen et al. 2002; Karin and Greten 2005; Scheidereit 2006). IKK is composed of two homologous kinases, IKK*α* (also known as CHUK) and IKK*β* (also known as IKBKB), each with potentially distinct roles in signal transduction (DiDonato et al. 1997; Mercurio et al. 1997, 1999; Hacker and Karin 2006; Scheidereit 2006; Hayden and Ghosh 2008). These two molecules attach to a multiprotein complex that requires NEMO (NF‐kB essential modulator; also known as IKBKG, IKK*γ*), heat‐shock protein‐90 (HSP‐90) and other proteins (Yamaoka et al. 1998; Chen et al. 2002; Broemer et al. 2004; Verma et al. 2004; Pittet et al. 2005; Fontan et al. 2007; Hinz et al. 2007). In addition, because IKK*α* and IKK*β* can both be found in complexes that weigh from 300 to 900 kDa, investigators have improved fractionation and genomic approaches to identify the individual components of the “IKK signalosome” (DiDonato et al. 1997; Mercurio et al. 1999; Chen et al. 2002; Broemer et al. 2004). This variability in complex size suggests that IKK*α* and IKK*β* may form a variety of predecessor and recycled complexes, besides the active one (Hacker and Karin 2006; Scheidereit 2006; Hinz et al. 2007; Hayden and Ghosh 2008).

The 180‐kD clathrin heavy chains (CHC) are present in abundance in all cells and can assemble into an extended variety of shapes and sizes to interact with proteins during endocytosis and exocytosis. However, CHC complexes can influence the signal trajectory for several receptors toward apoptosis and MAPK recruitment (Pierce et al. 2000; Rakhit et al. 2001; Schneider‐Brachert et al. 2004; McLaughlin et al. 2006, 2008). We have previously reported that silencing CHC with specific small interfering RNAs (siRNA) significantly attenuated TNF*α*‐induced phosphorylation of I*κ*Bα (Escobar et al. 2006). Depleting CHC also decreased the production of NF‐kB‐regulated MCP1 (monocyte chemoattractant protein 1), ICAM1, and phosphorylation of p65 Rel A (another recognized IKK activity; Escobar et al. 2006). Interestingly, CHC silencing also appeared to affect levels of phosphorylated I‐κB and p65 NF‐κB compared to unstimulated control cells (Escobar et al. 2006). Recently, Kim et al. (2011) have found that CHC regulates basal IKK*α* activity in unstimulated (resting) epithelial cells. The interaction appeared independent of the clathrin light chain or endocytosis. We hypothesized that clathrin forms complexes with both IKK*α* and IKK*β* in resting cells and sought (i) to detect the complexes by affinity pull‐down and MS proteomics and (ii) to localize their intracellular distribution by fluorescent immunostains and FRET.

## Methods

### Cell culture

Human lung epithelial cells (A549) were grown in Modified F12 media (Mediatech, Herndon, VA) enriched with 10% fetal bovine serum (FBS; Mediatech) and 100 IE/mL penicillin and 0.1 mg/mL streptomycin (Mediatech) in a hydrated incubation chamber kept at 37°C with 5% CO_2_.

### Flow cytometry

Isolated A549 cells, in suspension (1 × 10^6^ cells), were fixed and permeabilized with acetone/methanol (70/30) at −20°C for 10 min. Cells were washed three times with cold PBS. The cells were then incubated with 10% normal donkey serum in PBS for 1 h at room temperature to block nonspecific serum binding sites. After removal of the blocking serum, cells were incubated with primary antibodies overnight at 4°C. Excess antibody was removed by three washes with cold PBS. Fluorophore‐conjugated, species‐specific donkey secondary antibodies were added for 1 h at room temperature. After removal of excess antibodies by three washes with cold PBS, cells were resuspended in a 4% PFA‐PBS solution and analyzed on a Beckman FC500 flow cytometer within 24 h.

### Coimmunoprecipitation (IP)

Cells were grown in 100‐mm dishes until reaching 90–100% confluence and rinsed twice with 1 mL PBS per well at room temperature. Cells were lysed by adding 1 mL/dish of M‐PER lysis solution (Pierce Biotechnology, Inc., Rockford, IL) at room temperature for 5 min and then scraped with a rubber policeman into 2‐mL Eppendorf tubes and centrifuged at 14,000 RPM for 10 min to eliminate particulates. Each IP tube received 400 *μ*L of cell lysate with 2 *μ*g of each IKK antibody conjugated to agarose. The corresponding conjugated isotype or PBS (40 *μ*L) was used as controls. The incubations were carried at room temperature for 1 h and the pellets underwent five cycles of washing with 1 mL PBS. The final bead pellets were resuspended in 150 *μ*L PBS with 50 *μ*L 4 × Laemmli digestion buffer (Pierce Biotechnology, Inc.), boiled for 5 min, and spun at 5000 g for 10 min. The bead pellets were discarded and the supernatants were processed for mass spectrometry and proteomic analysis.

The IP antibodies used were as follows: agarose‐conjugated mouse anti‐IKK*α* and rabbit anti‐NEMO (Santa Cruz Biotechnology, Inc, Santa Cruz, CA); agarose‐conjugated mouse IgG and rabbit IgG (Santa Cruz Biotechnology, Inc) were used as nonspecific controls.

### Liquid chromatography–tandem mass spectrometry

The SDS–polyacrylamide gel electrophoresis was performed using 4−12% Bis Tris gel system (Invitrogen‐Novex, Carlsbad, CA) according to the manufacturer's protocol. The gel was stained with Coomassie brilliant blue stain (Invitrogen) and slices of equal size were excised from each sample lane, reduced using 10 mmol/L DTT at 65°C for 45 min, alkylated with 55 mmol/L iodoacetamide for 0.5 h at ambient temperature in the dark and digested in‐gel with sequencing grade porcine trypsin (Promega, Madison, WI) overnight at 37°C. Peptides were extracted three times from the gel using 50% ACN, 1% FA, concentrated by SpeedVac to a desired volume (~16 *μ*L), and subjected to LC‐MS/MS analysis.

Nano‐flow reverse phase LC‐MS/MS was performed using a capillary HPLC system (Agilent 1200, Palo Alto, CA) coupled with a linear ion trap mass spectrometer LTQ‐FT Ultra Hybrid ion cyclotron resonance mass spectrometer (Thermo Fisher; San Jose, CA) through an in‐house built nanoelectrospray ionization source. Tryptic peptides were preconcentrated and desalted onto a C_18_ trap column ZORBAX 300SB‐C_18_, (5 *μ*m i.d. × 5 mm; Agilent Technologies, Santa Clara, CA) with 5% ACN, 0.1% FA at a flow rate of 15 *μ*L/min for 5 min. The separation of the tryptic peptides was performed on a C_18_ reverse phase column (75 *μ*m ID × 360 *μ*m OD × 100 mm length) packed in‐house with 4 *μ*m 100 Å pore size C_18_ reversed‐phase stationary phase (Synergy; Phenomenex, Torrance, CA) kept at a constant 40°C using an in‐house built column heater at a flow rate of 380 nL/min. The mobile phases consisted of 5% acetonitrile with 0.1% formic acid (A) and 95% acetonitrile with 0.1% formic acid (B), respectively. A 90‐min linear gradient from 5 to 50% B was typically used. Data acquisition was performed using the instrument supplied Xcalibur (version 2.0.6, Thermo Scientific, San Jose, CA) software. The LC runs were monitored in positive ion mode by sequentially recording survey MS scans (m/z 400–2000), in the ICR cell, while three MS2 were obtained in the ion trap via CID for the most intense ions.

### Database searching, protein identification

Peptide identification was carried out using MASCOT server (Version 2.2, Matrix Sciences Ltd, London, U.K.) for MS/MS spectra assignment to the *Homo sapiens* subset of the SwissProt database. Peptide tolerance was set at ± 10 ppm with MS/MS tolerance set at ±0.6 Da. Trypsin specificity was used allowing for 1 missed cleavage. The modifications of Met oxidation, protein N‐terminal acetylation, and peptide N‐terminal pyroglutamic acid formation were allowed for, and Cys carbamindomethylation was set as a fixed modification. The results were exported into Scaffold (Proteome Software, Proteome Software, Inc, Portland, OR) that was used to filter and compare MS/MS‐based peptide and protein identifications. Peptide identifications were accepted if they could be established at >95.0% probability as specified by the Peptide Prophet algorithm. Protein identifications were accepted if they could be established at >99.0% probability and contained at least two identified unique peptides. The results were converted into txt files for Fold Change calculation (FC score) and Significance Analysis of INTeractome (SAINT) scoring http://www.ncbi.nlm.nih.gov/pubmed/22948729 and http://www.ncbi.nlm.nih.gov/pubmed/21131968 to generate a ranked list of putative interactors using the tool on the CRAPome.org site (Mellacheruvu et al. 2013).

Affinity controls deposited in CRAPome.org used were all entries in the database that used agarose as the affinity support, were from HEK293, HeLa and Jurkat cells, and used anti‐GFP or IgG as the affinity reagent. These parameters resulted in 11 controls (CC42, 44, 45, 46, 47, 48, 195, 196, 197, 198, and 199) used for this analysis. FC‐A scoring parameters: user controls, default background estimation, and average combining of replicates. FC‐B scoring parameters: all controls (user & CC), stringent background estimation, and geometric combining of replicates. SAINT scoring parameters: User controls, n‐burn 2000, n‐iter 4000, LowMode 0, MinFold 0, Normalize 1, and geometric combining of replicates. As shown in Table 1, protein rank was generated by summing the two spectral counting methods (FC_A, FC_B) with the SAINT score (×100).

**Table 1.** Proteomic analysis of immunoprecipitates from IKKα and NEMO pull downs. Top 20 proteins identified at high significance in each set of immunoprecipitates.

RANK	PROTID	GENE	FC A	FC B	SAINT
IKKA					
1	IKKA_HUMAN	*CHUK*	80.43	63.18	1
2	IKKB_HUMAN	*IKBKB*	72.01	57.06	1
3	NEMO_HUMAN	*IKBKG*	51.84	41.22	1
4	ILF2_HUMAN	*ILF2*	3.98	0.58	1
5	TCP4_HUMAN	*SUB1*	3.23	2.75	1
6	CLH1_HUMAN	*CLTC*	7.25	5.57	1
7	DHX9_HUMAN	*DHX9*	6.77	2.35	1
8	RS16_HUMAN	*RPS16*	3.48	0.82	1
9	TSP1_HUMAN	*THBS1*	17.23	13.41	1
10	SEC13_HUMAN	*SEC13*	13.09	10.41	1
11	MYO1B_HUMAN	*MYO1B*	6.41	4.27	1
12	FINC_HUMAN	*FN1*	4.85	3.95	1
13	MYO1E_HUMAN	*MYO1E*	3.53	2.77	1
14	HNRPC_HUMAN	*HNRNPC*	2.38	1.01	0.99
15	MTMRD_HUMAN	*SBF2*	5.11	3.86	0.99
16	TMEDA_HUMAN	*TMED10*	3.61	3.01	0.99
17	SSA27_HUMAN	*SSSCA1*	3.43	2.88	0.99
18	DAZP1_HUMAN	*DAZAP1*	3.6	2.93	0.98
19	TIM50_HUMAN	*TIMM50*	3.65	2.79	0.98
20	TFG_HUMAN	*TFG*	2.77	2.44	0.97
NEMO					
1	IKKB_HUMAN	*IKBKB*	65.13	50.75	1
2	IKKA_HUMAN	*CHUK*	63.23	50.22	1
3	NEMO_HUMAN	*IKBKG*	62.59	49.37	1
4	CO3_HUMAN	*C3*	17.49	12.61	1
5	TERA_HUMAN	*VCP*	22.5	9.43	1
6	PABP1_HUMAN	*PABPC1*	16.12	4.08	1
7	TFG_HUMAN	*TFG*	4.55	4.04	1
8	ILF2_HUMAN	*ILF2*	5.68	0.82	1
9	RBM14_HUMAN	*RBM14*	5.17	0.3	1
10	ROA2_HUMAN	*HNRNPA*	6.31	1.47	0.99
11	TCP4_HUMAN	*SUB1*	5.3	3.78	0.98
12	RBP56_HUMAN	*TAF15*	4.51	3.56	0.98
13	FUS_HUMAN	*FUS*	4.97	2.93	0.98
14	HNRL1_HUMAN	*HNRNPU*	3.31	2.64	0.98
15	HNRH3_HUMAN	*HNRNPH*	3.39	2.68	0.97
16	EWS_HUMAN	*EWSR1*	6	2.17	0.97
17	ROA3_HUMAN	*HNRNPA*	3.88	1.5	0.96
18	HNRH1_HUMAN	*HNRNPH*	2.6	2.24	0.95
19	RO52_HUMAN	*TRIM21*	1.38	1.04	0.9
20	CLH1_HUMAN	*CLTC*	8.52	5.63	0.88

### Immunofluorescent microscopy and FRET analysis

Cells were grown to 70–80% confluence on glass slides and were washed with phosphate‐buffered saline solution (PBS) prior to being fixed and permeabilized with 70/30 acetone/methanol solution. They were then treated with 10% normal donkey serum (Jackson Immunologicals, Westgrove, PA) in PBS for 1 h, followed by primary antibodies to IKK*α*, IKK*β*, NEMO, CHC, HSP‐90 (Santa Cruz Biotechnology, Inc) or isomolar, species‐specific IgG (Pierce Biotechnology, Inc.) and left overnight at 4°C in a humid slide chamber. The slides were washed three times with PBS and the cells were incubated for 1 h at room temperature with the following fluorochrome‐conjugated secondary antibodies: donkey anti‐rabbit‐Cy5, donkey anti‐goat‐Cy5and donkey anti‐mouse Cy3 (Jackson Immunologicals) or donkey anti‐rabbit Alexa 488, and donkey anti‐goat‐Alexa 488 (Invitrogen‐Molecular Probes, Carlsbad, CA) as appropriate (Mandal et al. 2008). Nuclei were stained with bis‐Benzamide (Sigma, St. Louis, MO).

Fluorescent resonance energy transfer (FRET) images were acquired using a Marianas imaging station (Intelligent Imaging Innovations, Denver CO) based on a Zeiss 100 m Axiovert microscope, using a Zeiss 63× Plan‐Apochromat objective (1.4 N/A), a Sutter Xenon light source and a Cooke SensiCam (1376 × 1040 pixel resolution, The Cooke Corporation, Romulus, MI, USA). Chroma Sedat filter sets with single emission and excitation filter, and a multiband pass dichroic, were used for emission detection. To obtain FRET images, a Z‐plane stack with >20 planes at 0.2 *μ*m was acquired and images were processed using a constrained iterative deconvolution algorithm based upon acquisition‐specific point‐spread functions. For each Z‐stack, six channels were captured with the same exposure times (except the nuclear stain). The following filter configurations were used for image capture and FRET analysis: Bis‐benzimide (ex – S403/12x, em – S457/50 m), Alexa 488 (ex – S490/20x, em – S528/38 m), Cy3 (ex – S555/28x, em – S617/73 m), Cy5 (ex – S625/20x, em – S685/40 m), Alexa 488:Cy3 FRET (ex – S490/20x, em – S617 m), Cy3:Cy5 FRET (ex – S555/28x, em – S685/40 m). The corrections for bleed through (Gordon et al. 1998; Berney and Danuser 2003) were done by imaging and processing slides that were incubated with a single fluorophore, under the same conditions as the experimental group, and using an automated operation within Slidebook. The bleed‐through coefficients were as follows: Alexa 488‐Cy3 FRET pair: Alexa 488‐0.105, Cy3‐0.2; Cy3‐Cy5 FRET pair: Cy3‐0.034, Cy5‐0.078.

Images were masked to select the voxel intensities that were above nonspecific binding (obtained with isotypes at the same concentrations as the primary antibodies). Masks were created for individual fluorophores (Alexa 488, Cy3 and Cy5) and for the fluorescence energy transfer detected between the FRET pairs (Alexa 488:Cy3, Cy3:Cy5). Mask operations calculated voxels containing donor, acceptor and positive transfer channels. The corrected FRET (cFRET) was calculated as cFRET = Transfer‐Fd/D donor‐Fa/Aa acceptor as reported (Gordon et al. 1998; Berney and Danuser 2003). The intensity of the positive voxels in any given cell area is reported in linear pseudocolor where black is “cold” (no cFRET) and red is “hot” (high cFRET voxels). A legend demonstrating these results is displayed on each image.

To further control for false‐positive FRET, each result was tested with two different secondary antibodies to allow us to determine significant cFRET signal for each protein pair when the labeling secondary was either a donor, or an acceptor. Thus, if cFRET is reported herein, it has been found to be present regardless of the fluorescently labeled secondary antibody used; likewise, protein pairs reported to be negative for cFRET did not have a positive result in either combination in areas of overlap.

### FRET resolution measure

Under ideal conditions of appropriate spectral overlap and averaged dipole orientation, FRET between fluorescent dyes in aqueous media is 50% at 5–6 nm distance (Berney and Danuser 2003; Lakowicz 2006). In select cases, FRET measurements can establish distance estimates of molecular proximity. Unfortunately, a lack of FRET signal does not necessarily reflect lack of proximity between epitopes In order to span larger molecular complexes, we and others have used antibodies (mean epitope to fluorophore spacing of about 6 nm) instead of direct labeling with dyes or fluorescent protein constructs (<2 nm; Konig et al. 2006; McLaughlin et al. 2006, 2008; Wei et al. 2006; Mandal et al. 2008). Although the theoretical resolution of neighboring epitopes could be degraded to ~30 nm (2 × 12 nm the maximum possible length of two primary/secondary antibody pairs, plus the interfluorophore distance), this is an improvement over the best possible deconvolved optical resolution by 5‐ to 10‐fold (where z resolution is ~130 nm). Longer spacer arms also provide conformational flexibility purveying a much wider range of favorable fluorophore orientations, even as the degraded distance resolution is still acceptable for detecting large oligomeric conglomerates (Konig et al. 2006; Wei et al. 2006).

Voxel counts have been used to determine equilibration kinetics across compartments in cells. The calculated resolution of *xyz* dimensions of voxels obtained in the current conditions (based on objective, NA, point spread function) are around 10^2^ × 10^2^ × (2 × 10^2^) nm. Therefore, the density of individual 5–20 nm sized protein complexes could be 10–50 units, along the diagonal of the voxel. Thus, the overlap of FRET pairs in a voxel suggests congestion of different binary complexes that are perhaps no more than a few dozen molecular diameters away.

The number of voxels satisfying a threshold signal can reflect the localized amount of the species of interest across any cell, while the intensity remains a more complex function. As in conventional immunofluorescent microscopy, the recorded intensity of a voxel in FRET images is the summed emission from fluorophores. Thus, the voxel's FRET intensity depends on the number of molecules of the dye in that volume, plus the distribution of FRET efficiencies for individual excitation pairs. Other physical factors, such as quantum yields of fluorophores and refractive indices, are presumed constant for group comparisons (assuming uniform labeling across cells and in replicate experiments).

### Data analysis

All experiments were repeated at least three times. Data are presented as mean ± SEM. Comparisons between two groups were assessed by *t*‐test, and those among three or more groups were assessed by analysis of the variance using JMP 5.0 software (SAS Institute, Inc. Cary, NC USA), and presented with Microsoft Excel 2003 graphs. We accepted statistical significance for values of *P* < 0.01. Mean fluorescent intensities were determined with the Intelligent Imaging Innovations Slidebook 4.1 software (Intelligent Imaging Innovations, Denver, CO).

## Results

### CHC immunoprecipitates with IKK*α* and NEMO

Using commercially available agarose‐conjugated antibodies for affinity pull down, MS analysis revealed at least 91 proteins that produced at least two identifiable fragments with the anti‐IKK*α* antibody and over 200 with the anti‐NEMO antibody in at least one replicate run. The top three proteins in both pull downs were the canonical members of the IKK signalosome. The isotype antibody pull down produced no hits for these in any run, leading to high scores for both FCa and FCb (Table 1). We did not use commercially available agarose‐conjugated IKK*β* antibody for pull downs because it is directed to an epitope at the C‐terminus of the protein, which could be sterically obstructed by bound NEMO (Fontan et al. 2007; Hayden and Ghosh 2008). The clathrin heavy chain 1 (also known CLH1, CLTC, and CHC), the focus of this study, emerged as one of the top 20 partners of IKK*α* and NEMO (Table 1). Three other proteins (ILF2, SUB1, and TFG) appear on both pull‐down interactomes. The functions of these proteins and their putative partners can be found on the curated NCBI website. TFG, a TRK‐fused gene, has been directly linked to activation of the NF‐kB pathway. ILF2 is one of the proteins making up the heterodimeric NFAT transcription factor. The complex has been shown to repair DNA breaks and may also negatively regulate micro‐RNA processing. SUB1 is also involved with DNA repair. SUB1 may play complex roles in the steps of gene expression affecting initiation, elongation, termination, and re‐initiation by RNA polymerase. Curiously, many proteins in both interactomes appear to be involved with RNA processing. For example, interacting with IKK*α* are DHX9, an RNA helicase, and RS16 a component of the 40S subunit of the ribosome. With NEMO, PABPC1 is a polyA‐binding protein, while RBM14 is a ribonucleoprotein that acts as an RNA splicing modulator. Over six members of the heteromolecular human ribonuclear protein complex were detected with high confidence in both interactomes. This despite the fact that these proteins appear regularly in the CRAPome with agarose‐conjugated antibodies and are discriminated severely by the FCb score. In our experiments, we found no peptide fragments when using agarose‐conjugated irrelevant antibodies, suggesting novel RNA linked processes, unrelated to NF‐kB activation or clathrin. Remarkably, none of the well‐known partners of clathrin involved in endocytosis, including the stoichiometric clathrin light chain, were ever encountered. However, two myosin motor isoforms appear in the top 20 interactome s of IKK*α* and NEMO.

Of previously described partners, a few fragments of HSP90 beta were found but did not achieve significance over isotype controls, whereas Cdc37 (Chen et al. 2002; Hinz et al. 2007) or ELKS (Ducut Sigala et al. 2004) were never detected. This illustrates that affinity pull down may miss well‐known complexes such as the one with HSP90 (Chen et al. 2002; Broemer et al. 2004; Pittet et al. 2005; Hinz et al. 2007; see figs below).

### Cellular contents by flow analysis and distributions of IKK*α* and IKK*β* by digital microscopy and FRET

**Figure 1.**
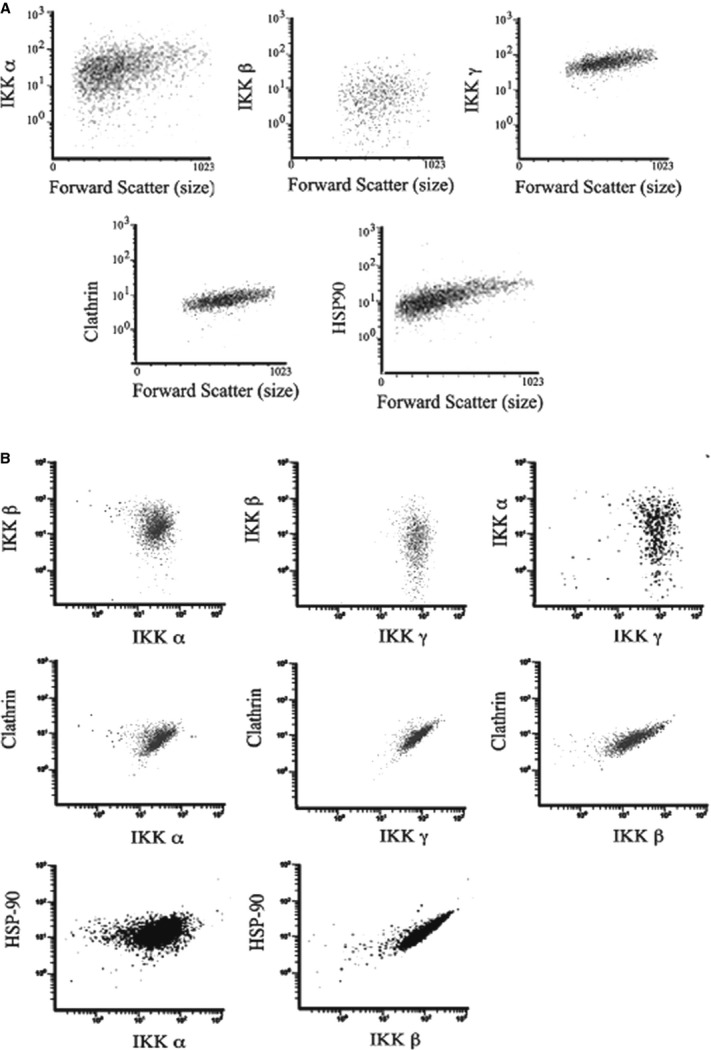
Widely varying contents of IKK*α* and IKK*β*, but not HSP90 and CHC proteins, in resting cells (A). Representative cytometric plots of the Mean Fluorescence Intensity (MFI) of IKK*α* and IKK*β* are poorly correlated with cell size (forward scatter) and each other (B), while NEMO, CHC, and HSP90 increase monotonically.

As a preliminary, cells were analyzed by flow cytometry to examine the relative intracellular content of IKK*α*, IKK*β*, IKK*γ*, Hsp90, and clathrin. The cellular content of IKK*α* and IKK*β* correlated poorly with cell size (forward scatter), disparate from the intracellular distribution of clathrin or Hsp90. A systematic multichannel flow cytometric analysis of cells simultaneously stained with two or three fluorophores showed variations of almost 100‐fold for IKK*α* and IKK*β* in individual cells and less so for clathrin, NEMO or HSP90 (Fig. 1A). Furthermore, correlation of the cellular content of IKKs with each other and the other members of the canonical complex showed that IKK*α* and IKK*β* levels were not correlated with one another, but were somewhat better related to CHC or Hsp90 content (Fig. 1B).

Therefore, to better understand how complexes are formed in cells from proteins with varying individual amounts, FRET analyses of intact cells were completed with special attention to detailing the percentage of voxels of FRET for discrete cellular locations. We anticipated that a large percentage of the FRET interactions among proteins would likely dictate that such interactions are important, whereas those interactions which comprise only a minority of the proteins of interest are likely to be of little intrinsic value.

A few studies have spatially located the principal IKK proteins (*αβ*) individually (Birbach et al. 2002; Verma et al. 2004; Ear et al. 2005; Harhaj et al. 2007) but the intracellular locations of the IKK complex, together and with clathrin, have not been studied. Figures 3–8 show deconvolved z‐projections of representative single cells. The panels depict combinations of CHC localized with the two IKK proteins, NEMO and Hsp90 as detected by indirect fluorescence with simultaneous, four‐channel, three‐dimensional imaging (Cy5, Cy3, ALEXA 488‐labeled anti‐species Ig; nuclear DNA in blue) of intact cells. The corresponding cFRET observed between channel pairs is always shown at right, on a linear colored scale. The spots represent pairs of epitopes clustered within 30 nm and could include from binary up to larger heteromeric complexes. In comparison, the extended CHC is itself about 47.5 nm long (Brodsky et al. 2001; Fotin et al. 2004), well beyond the 5–6 nm range of interdye FRET (Lakowicz 2006). The superimposition of cFRET pairs (red, green), and the dependence of overlap on each cFRET pair (regressions, *n* = 27), is shown throughout.

The magnitude of cFRET depends on the ratios of donor–acceptor pairs, ensuring a comparable fluorescent intensity at the CCD. By optimizing labeling titers, we can obtain high FRET efficiencies (Table 2) and reproducible results among replicate runs and with different batches of cells. Also, cFRET between labeled antibodies exploits pairs of modern fluorophore dyes that give minimal donor emission at the acceptor's emission wavelength, while retaining sufficient spectral overlap at the acceptor's excitation wavelength. Nonspecific fluorescence is controlled by prescripted fluorophore combinations controls, required for cFRET calculation (Gordon et al. 1998; Berney and Danuser 2003; Lakowicz 2006). This means corrections of 20% or less in the cFRET.

**Table 2.** Normalized FRET (FRETN; Gordon et al. 1998) of seven pairs of indirect immunostained proteins range from 0.3 to 0.7 (SEM) indicating that these clusters are robustly detected in cells in the basal state. Mean of four separate sets of experiments where the corrected FRET signal in each cell was normalized to the emission intensity of the acceptor.

Donor	Acceptor						
	IKK*β*	CHC	HSP90	IKK*α*	CHC	IKK*β*	HSP90
IKK*α*	0.362						
IKK*β*		0.522					
IKK*β*			0.632				
NEMO				0.326			
IKK*α*					0.70		
NEMO						0.426	
IKK*α*							0.30

**Figure 2.**
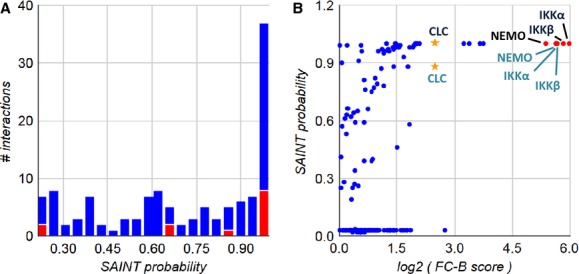
Probability of significance for proteins identified in IKK*α* and NEMO pull‐down experiments. (A) Histogram of SAINT probabilities for both data sets, proteins found in iRefIndex (protein–protein interaction database) are shown in red. (B) SAINT versus spectral counting (FC‐B) plot. IKK complex members, red dots; clathrin, stars; black text, identified from IKKa pull downs; blue text, identified from NEMO pull downs. SAINT scoring parameters: User controls, n‐burn 2000, n‐iter 4000, LowMode 0, MinFold 0, Normalize 1, and geometric combining of replicates. FC‐B scoring parameters: all controls (user & CC), stringent background estimation and geometric combining of replicates.

The overlap and dependence of IKK*α* and *β* with their chaperone Hsp90 appears as a cFRET+ signal within the nucleus and perinuclear arcs (Fig. 2; Broemer et al. 2004; Pittet et al. 2005; Qing et al. 2006; Hinz et al. 2007). IKK*α* fluorescence is more abundant in the nucleus than IKK*β*. Although Hsp90 is present in the nucleus, there is little IKK*β*–Hsp90 FRET within the nucleus. Both FRET pairs overlap at the perinuclear ring. A significant fraction of IKK*β*–Hsp90 cFRET voxels overlap IKK*α*–IKK*β* (0.55 ± 0.090) but considerably less IKK*α*–IKK*β* appears associated with Hsp90 (0.17 ± 0.03, Table 1). The amount of IKK*β*–Hsp90 cFRET signal strongly influences the degree of overlap (regression slopes = 0.55) despite the linear fit being poorer in this case (*r*^2^ = 0.66). The slope of the overlap against the IKK*α*–IKK*β* signal is weaker (0.26), but the linear fit is very high (*r*^2^ = 0.99). Importantly, the intercepts are negligible for both linear fits.

**Figure 3.**
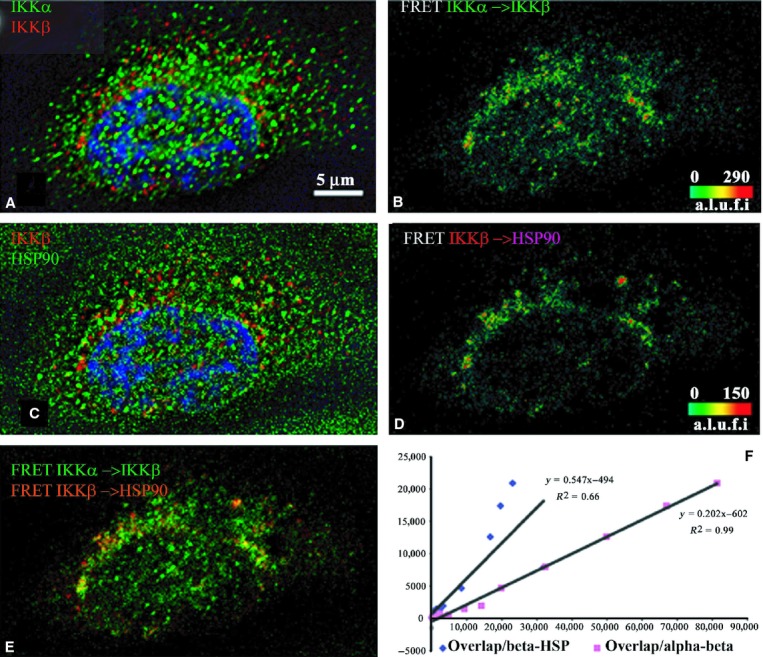
Indirect immunofluorescence detected cFRETs from IKK*α*–IKK*β* and IKK*β*–Hsp90, demonstrating the net overlap and the dependence of overlapped FRET on the amount of each cFRET in resting cells (*n* = 13). The top left panel (A) shows IKK*α* (green), IKK*β* (red), and nuclear stain in blue. Panel B shows the cFRET signal intensity pseudocolored from green (lowest) to red (highest). IKK*α*–IKK*β* cFRET is strong within the nucleus and around the perinuclear border. IKK*α* is more abundant in the nucleus than IKK*β*. The middle left panel (C) shows IKK*β* (red) and HSP90 (in Cy5, shown in green) while panel D shows the corresponding cFRET signal intensities ranging from green to red. Although Hsp90 is present in the nucleus, there is little cFREc from IKK*β* within the nucleus. Panel E shows both cFRET signals, from panel A (in green) and panel B (in red). Both FRET pairs overlap (yellow) modestly at the perinuclear edge and cytoplasm. (F) The graph shows that the number of voxels where both FRET signals overlap depends more on increasing numbers of voxels containing IKK*β*–HSP90 cFRET (slope = 0.547) than the number of voxels containing IKK*α*–IKK*β* cFRET (slope = 0.262) plotted from all examined cells.

The cFRET interactions of IKK*α* with IKK*β* are visualized at the broken perinuclear arcs (Fig. 3). Unlike Hsp90, CHC is essentially absent from the nucleus, though abundant in the perinuclear/Golgi area (Fig. 3). The cFRET+ interaction of the IKK*β*–CHC pair appears to be dictated by the absence of CHC in the nucleus; thus, the overlap of IKK*α*–IKK*β* with IKK*β*–CHC bears minimal overlap in the perinuclear and in the cytoplasmic compartments. As with Hsp90, a significant fraction of IKK*β*–CHC cFRET signal (0.5 ± 0.06) overlaps a small fraction of IKK*α*–IKK*β*, (0.26 ± 0.04). The slope of the overlap against the IKK*α*–IKK*β* signal is the weakest found (0.06), and the poor fit (*r*^2^ = 0.62) appears to be due to a complex distribution.

**Figure 4.**
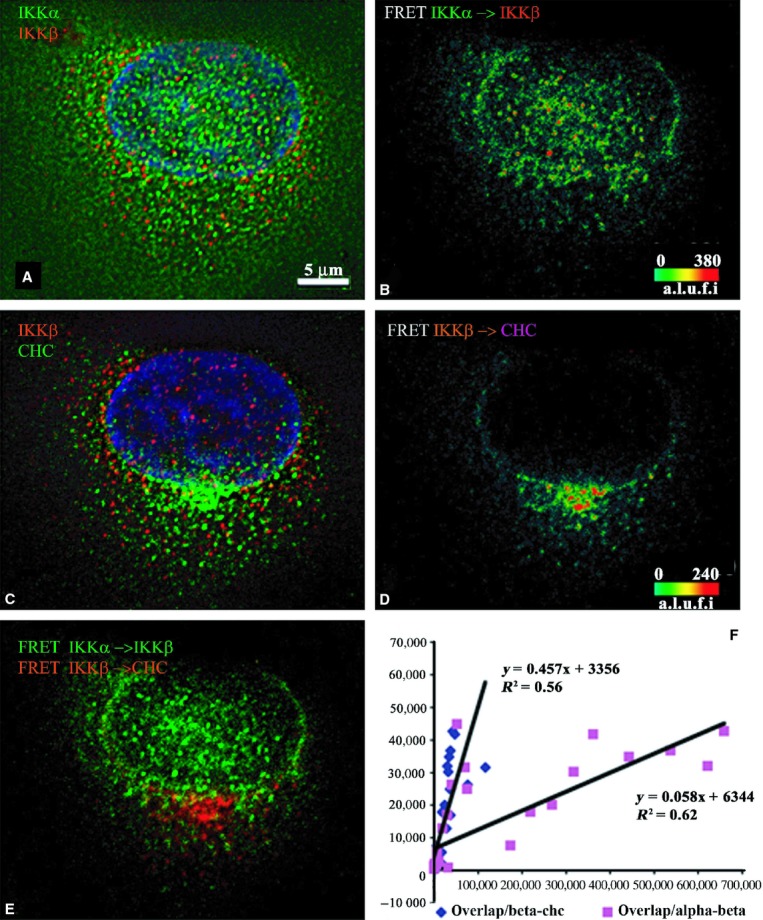
Indirect immunofluorescence detected cFRETs from IKK*α*–IKK*β* and IKK*β–*CHC, demonstrating the net overlap and the dependence of overlapped FRET on the amount of each cFRET in resting cells (*n* = 35). The top left panel (A) shows IKK*α* (green), IKK*β* (red), and nuclear stain in blue. Panel B shows the cFRET signal intensity pseudocolored from green (lowest) to red (highest). IKK*α*–IKK*β* cFRET is prominent within the nucleus and around the perinuclear border. The middle left panel (C) shows IKK*β* (red) and CHC (in Cy5, shown green) while panel D shows the corresponding cFRET ranging from green to red. cFRET from IKK*β*–CHC pairs appears to be dictated by the distribution of CHC, found mostly in cytoplasmic and peri‐Golgi areas. Panel E shows both cFRET signals, from panel A (in green) and panel B (in red). The overlap of IKK*α*–IKK*β* with IKK*β*–CHC complexes is minimal in perinuclear areas, but absent within the nucleus. (F) The graph shows that the number of voxels where both FRET signals overlap depends modestly on increasing numbers of voxels containing IKK*β*–CHC cFRET (slope = 0.457) but not on the number of voxels containing IKK*α*–IKK*β* cFRET (slope = 0.058) plotted from all examined cells.

In contrast, the CHC–IKK*β* and IKK*β*–Hsp90 cFRET signals are found in the perinuclear ring, but with substantial signal of the latter pair in the cytoplasm (Fig. 4). As with previous sets, there is little FRET positivity detected within the nucleus, while the CHC rich peri‐Golgi area is not as prominent here. A very significant fraction of the CHC–IKK*β* signal overlaps IKK*β*–Hsp90 FRET pixels (0.74 ± 0.07) and the linear dependence is almost a perfect one, the best correlation among the six triads (slope 0.96, *r*^2^ = 0.98). The dependence of the overlap on the amount of CHC–IKK*β* cFRET signal is also significant (0.41, *r*^2^ = 0.78) but not as high.

**Figure 5.**
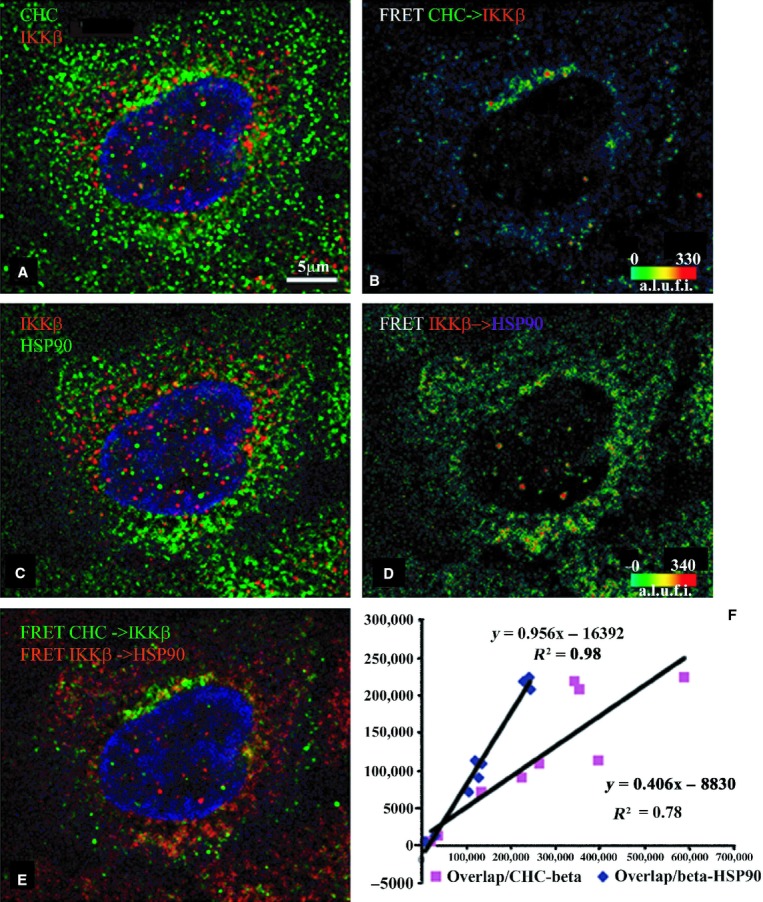
Indirect immunofluorescence detected cFRETs from CHC–IKK*β* and IKK*β*–HSP90, demonstrating the net overlap and the dependence of overlapped FRET on the abundance of each cFRET in resting cells (*n* = 9). The top left panel (A) shows CHC (green), IKK*β* (red) and nuclear stain in blue. Panel B shows the cFRET signal intensity pseudocolored from green (lowest) to red (highest). CHC–IKK*β* cFRET is present around the perinuclear border and cytoplasm. The middle left panel (C) shows IKK*β* (red) and HSP90 (in Cy5, shown green) while panel D shows the corresponding cFRET signal intensities ranging from green to red. cFRET from IKK*β*–HSP90 pairs is most abundant in cytoplasmic and perinuclear areas. Panel E shows both cFRET signals, from panel A (in green) and panel B (in red). (F) The graph shows that the amount of voxels where both FRET signals overlap depends almost exactly on increasing numbers of voxels containing IKK*β*–HSP90 cFRET (slope = 0.956), and modestly on the number of voxels containing CHC–IKK*β* cFRET (slope = 0.406) plotted from all examined cells.

The cFRET association of IKK*α*–CHC and IKK*α*–Hsp90 is strongest in the perinuclear ring but in different zones, such that overlap is minimal (Fig. 5). There are minimal IKK*α*–Hsp90 FRET pairs detected within the nucleus. While these features of IKK*α* FRETs are similar to IKK*β*, the dependence of the overlap of CHC‐IKK*α* on IKK*α*–Hsp90 complexes is not as dramatic (slope 0.54, *r*^2^ = 0.97). The significant and negative intercept (−2163, *P* = 0.0013) suggest that local concentrations of IKK*α*–Hsp90 appear before FRET pixels overlap with clusters of CHC–IKK*α*.

**Figure 6.**
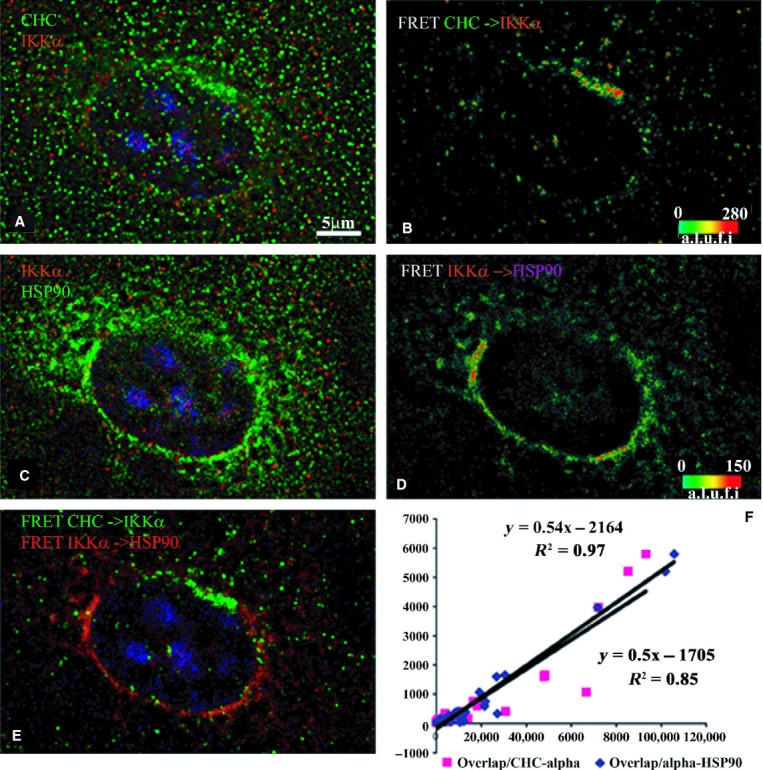
Indirect immunofluorescence detected cFRETs from CHC–IKK*α* and IKK*β*–HSP90, demonstrating the net overlap and the dependence of overlapped FRET on the amount of each cFRET in resting cells (*n* = 31). The top left panel (A) shows CHC (green), IKK*α* (red), and nuclear stain in blue. Panel B shows the cFRET signal intensity pseudocolored from green (lowest) to red (highest). CHC–IKK*α* cFRET is present around the perinuclear border and cytoplasm. The middle left panel (C) shows IKK*β* (red) and HSP90 (in Cy5, shown green) while panel D shows the corresponding cFRET signal intensities ranging from green to red. cFRET from IKK*α*–HSP90 pairs is most abundant in cytoplasmic and perinuclear areas, but virtually absent within the nucleus. Panel E shows both cFRET signals, from panel A (in green) and panel B (in red). (F) The graph shows that the amount of voxels where both cFRET signals overlap depends equally on increasing numbers of voxels containing IKK*α*–HSP90 cFRET (slope = 0.54), and the number of voxels containing CHC–IKK*α* cFRET (slope = 0.50), plotted from all examined cells.

In addition, the IKK*α*–CHC FRET pairs are less abundant than NEMO–IKK*α* FRET+ complexes, which show high efficiencies especially around the nucleus and cytoplasm (Fig. 6). Although both IKK*α* and NEMO are abundant in the nucleus, cFRET between these is rare in this compartment. Only about 8.1% ± 1.2% of NEMO–IKK*α* containing complexes overlap with IKK*α*–CHC voxels. The amount of voxels where both FRET signals overlap depends more on increasing numbers of voxels containing IKK*α*–CHC cFRET (slope = 0.48) than on the number of voxels containing NEMO–IKK*α* cFRET (slope = 0.15) plotted from all examined cells.

**Figure 7.**
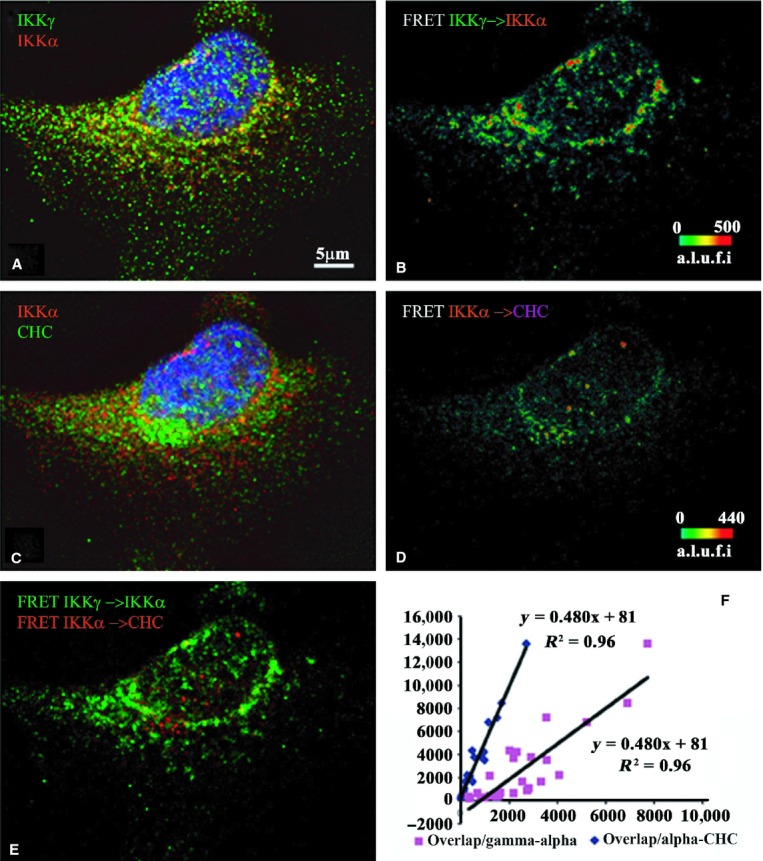
Indirect immunofluorescence detected cFRETs from NEMO–IKK*α* and IKK*α*–CHC, demonstrating the net overlap and the dependence of overlapped FRET on the amount of each cFRET in resting cells (*n* = 27). The top left panel (A) shows NEMO (green), IKK*α* (red), and nuclear stain in blue. Panel B shows the cFRET signal intensity pseudocolored from green (lowest) to red (highest). NEMO–IKK*α *cFRET is abundant around the perinuclear border, cytoplasm, and nucleus. The middle left panel (C) shows IKK*α* (red) and CHC (in Cy5, shown green) while panel D shows the corresponding cFRET signal intensities ranging from green to red. FRET from IKK*α*–CHC pairs is abundant in cytoplasmic and perinuclear areas. Panel E shows both cFRET signals, from panel A (in green) and panel B (in red). The graph shows that the number of voxels where both FRET signals overlap depends more on increasing numbers of voxels containing IKK*α*–CHC cFRET (slope = 0.48), and much less on the number of voxels containing NEMO–IKK*α* cFRET (slope = 0.15), plotted from all examined cells.

Finally, the binary NEMO–IKK*β* pairs show high cFRET signal in the cytoplasm and in particular perinuclear areas (Fig. 7). The NEMO–IKK*β* pairs overlap IKK*β*–CHC in similar cellular locales; however, although there is an abundance of NEMO, in the nucleus there is little cFRET with IKK*β*. The amount of voxels where both cFRET signals overlap depends more on increasing numbers of voxels containing IKK*β*–CHC cFRET (slope = 0.64) than on the number of voxels containing NEMO–IKK*β* cFRET (slope = 0.296).

Taken together, Figures 2–7 demonstrate that IKK*α* is more abundant within the nucleus than IKK*β*, although the IKK*α*–IKK*β* cFRET is clearly detected within nuclear voxels. Although NEMO is also found within the nucleus and cFRETs between NEMO and either IKK are detected elsewhere, there is scant IKK*α*–NEMO FRET and negligible IKK*β*–NEMO detected within the nucleus. These data support the hypothesis that NEMO plays different roles in the nucleus than in the cytoplasm (Verma et al. 2004; Ear et al. 2005; Gloire et al. 2006; Scheidereit 2006). Similarly, while Hsp90 is present within the nucleus, its complexes with IKK*α* or IKK*β* are restricted to arcs on the nuclear periphery. CHC is rare in the nucleus of these cells but indirect immunofluorescence with spacer antibodies detects cFRET with IKKs in the cytoplasm and perinuclear areas.

To summarize these data, the FRETn for all pairs examined was calculated and is shown in Table 2. The high values indicate that the ratio of donor and acceptors fluorescence intensity was close to 1 and that the concentration of FRET complex to free dye was satisfactorily high. Furthermore, the fraction of voxels containing two cFRETs and the regression of the overlap against the abundance of each pair has been complied for six pairs examined (Table 3). Unlike the mean fraction, which has high error, the regression uses the broad range of protein distribution in resting cells to define evidence of linear relationships with high accuracy. The greater slope dependence indicates the preferred direction of ternary overlap. These data indicate that neither IKK*β*–Hsp90 complexes nor IKK*β*–CHC complexes contribute significantly to the amount of IKK*α*–IKK*β* complexes. Instead, IKK*β*–CHC complexes seem proportionate to the quantity of IKK*β*–Hsp90 complexes and IKK–CHC complexes may precede IKK–NEMO complexes.

**Table 3.** Compiled statistics of fractions and regression slopes for all group studied for cFRET. Arrows represent the direction of energy transfer from donor to acceptor.

Overlap	IKK*α*→IKK*β* ∪ IKK*β*→HSP90		IKK*α*→IKK*β* ∪ IKK*β*→CHC		CHC→KK*β* ∪ IKK*β*→HSP90		CHC→IKK*α* ∪ IKK*α*→HSP90		NEMO→IKK*α* ∪ IKK*α* →CHC		NEMO→IKK*β* ∪ IKK*β*→CHC	
Fraction of	IKK*α*→IKK*β*	IKK*β*→HSP90	IKK*β*→CHC	IKK*α*→IKK*β*	CHC→IKK*β*	IKKB→HSP90	CHC→IKK*α*	IKK*α*→HSP90	IKKg→IKK*α*	IKK*α*→CHC	IKKg→IKK*β*	IKK*β*→CHC
Mean	0.171	0.546	0.497	0.256	0.409	0.740	0.391	0.336	0.081	0.504	0.217	0.4052
Standard ERROR	0.029	0.089	0.057	0.036	0.049	0.067	0.039	0.035	0.012	0.025	0.017	0.032
99% confidence interval	0.08 –0.26	0.28 –0.81	0.34 –0.65	0.16 –0.36	0.24 –0.57	0.51 –0.97	0.28 –0.50	0.24 –0.43	0.05–0.12	0.43–0.57	0.17 –0.26	0.32 –0.49
Linear regression on	IKK*α*→IKK*β*	IKK*α*→HSP90	IKK*β*→CHC	IKK*α*→IKK*β*	CHC→IKK*β*	IKKB→HSP90	CHC→IKK*α*	IKKA→HSP90	NEMO→IKK*α*	IKK*α*→CHC	NEMO→IKK*β*	IKK*β*→CHC
*R* ^2^	0.99	0.66	0.56	0.62	0.78	*0.98*	*0.85*	0.97	*0.76*	0.96	*0.94*	*0.95*
Slope	0.26	0.55	0.46	0.058	0.406	0.96	0.50	0.54	0.15	0.48	0.296	0.640
*P* value	1.14E‐12	0.000793	3.34E‐07	2.46E‐08	0.001557	5.85E‐07	2.3E‐13	1.1E‐22	2.71E‐09	3.43E‐19	3.73E‐16	1.23E‐16
Intercept	−602.3	490.5	3355.9	6343.6	8829.2	−16,392.2	−1704.92	−2163.6	−1365.66	81.1	−892.5	−1611.2
*P* value	0.02	0.77	0.18	**0.004331**	0.74	0.11	0.193	**0.001377**	0.017	0.61	0.074	**0.002652**

Bold numbers indicate significance at *P* > 0.005.

## Discussion

Previous data demonstrated that silencing clathrin alters I‐kB phosphorylation and NF‐kB activation in both unstimulated and stimulated A549 lung epithelial cells and that clathrin must be involved in either the activatory signaling pathway or in housekeeping cycles of the resting state (Escobar et al. 2006). In the current study, quantitative evidence (proteomics and first‐order regression of bimolecular complexes against cytometric abundance) showed that clathrin is involved with elements of the IKK signalosome operating in the unstimulated state of a human Type II alveolar cell line.

The data from these two reports provide independent support for recent discoveries concerning the multifaceted roles of the dynamic IKK complex. In addition to regulating the NF‐kB pathway, the IKK proteins play other roles in immunity, growth, and cancer and although the structural components of the active IKK signalosome (*α*,* β*, NEMO) are known, the maturation and posttranslational processing are just being explored (Hacker and Karin 2006; Scheidereit 2006; Hayden and Ghosh 2008). IKK*α–*IKK*β* and NEMO are known to be multifunctional proteins in both cell cytoplasm and nucleus (Gloire et al. 2006; Scheidereit 2006; Hinz et al. 2007) and flow cytometric analysis showed variation of almost 100‐fold for IKK*α* and IKK*β* in individual cells, less so for clathrin, NEMO or HSP90 (Fig. 1A).

**Figure 8.**
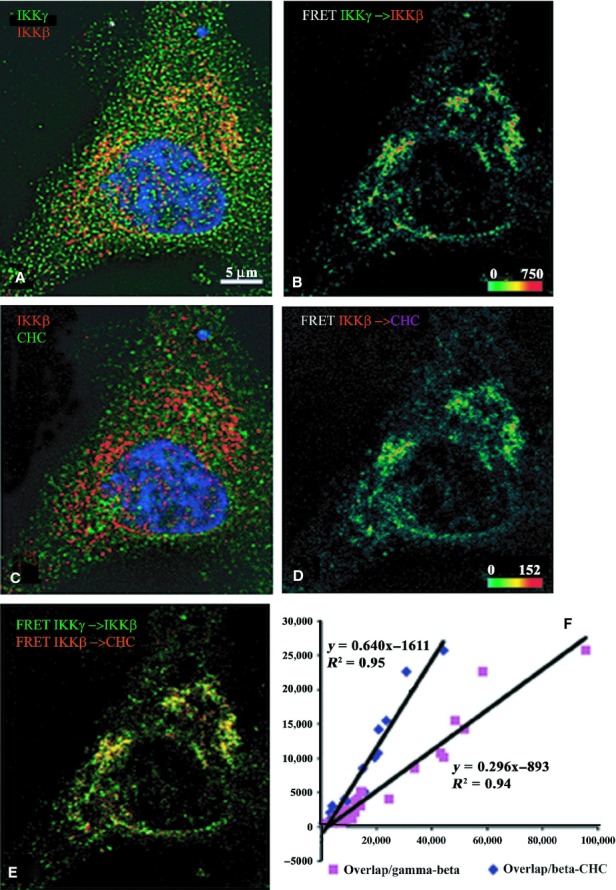
Indirect immunofluorescence detected cFRETs from NEMO–IKK*β* and IKK*β*–CHC, demonstrating the net overlap and the dependence of overlapped FRET on the amount of each cFRET in resting cells (*n* = 26). The top left panel (A) shows IKK*γ* (green), IKK*β* (red), and nuclear stain in blue. Panel B shows the cFRET signal intensity pseudocolored from green (lowest) to red (highest). NEMO‐IKK*β* cFRET is abundant around the perinuclear border and cytoplasm, but not within the nucleus. The middle left panel (C) shows IKK*β* (red) and CHC (in Cy5, shown green) while panel D shows the corresponding cFRET signal intensities ranging from green to red. cFRET from IKK*β*–CHC pairs is also abundant in cytoplasmic and perinuclear areas, but virtually absent within the nucleus. Panel E shows both cFRET signals, from panel A (in green) and panel B (in red). (F) The graph shows that the number of voxels where both cFRET signals overlap depends more on increasing numbers of voxels containing IKK*β*–CHC cFRET (slope = 0.64), and less so on the number of voxels containing NEMO–IKK*β* cFRET (slope = 0.296), plotted from all examined cells.

Cross‐correlating the cellular contents of these five proteins with one another revealed distributions approximating linear relationships between clathrin–NEMO, clathrin–IKK*β* and HSP90–IKK*β*. We hypothesized that this substantial variation could be exploited to quantify how the amounts of these protein pairs influenced the overlap with other pairs within single cells. Integrated FRET in individual cells (Figs 3–8) shows variation over almost five logs for certain intracellular pairs, including NEMO–IKK*β* or IKK*α*–IKK*β* and more restricted ranges for the total cellular FRET between IKK*β*–Hsp90 and IKK*β*–CHC.

Scheidereit (2006) reviewed a large number of proteins that could potentially interact with IKK*α*–IKK*β* and NEMO and concluded that the presence of partner proteins probably depended on the cell type studied and on the stimulus used, if any (Scheidereit 2006).

Many of the studies on the IKKs, though, used labeled proteins and none have investigated the human lung epithelium‐derived cell types.

In this study, no fragments of clathrin vesicle associated proteins were found with the IKKs. This finding supports previous data, which contended that the involvement of clathrin with IKK*α* signaling in resting epithelial cells is independent of the endocytic pathways (Kim et al. 2011).

The contaminant repository shows that affinity pull down with agarose‐conjugated beads (chiefly in HeLa, Jurkat, T‐cells) often finds components of the cytoskeleton, ribosomal proteins, histones, and keratins (Mellacheruvu et al. 2013). Thus, it is surprising that the SAINT analysis finds human ribonucleoprotein C (HNRNPC) in IKK*α* pull downs with high confidence; this protein could be a likely IKK*α* partner in these cells, since the isotype IgG pull downs found very few peptide fragments of the protein. Furthermore, other components of this large ribonuclear complex, such as HNRNPK, HNRNH3, HNRNPF, HNRNPU, were found with significance >0.5 (data uploaded to CRAPOME).

The roles of IKK*α* and NEMO in the nucleus have been scrutinized (Gloire et al. 2006) using cell disruption approaches. Birbach et al. (2002) were the first to image IKK*α* (and less IKK*β*) by immunostain within the nucleus, with a larger nuclear population of IKK*α* in an endothelial cell line than in HELA cells (which required blockade of nucleocytoplasmic transport for detection). Interestingly, they noted that direct YFP, GFP‐labeled IKK*α* constructs translocated a little slower than the native proteins, perhaps because of the presence of the tag increasing their size. In addition, Sil et al. (2004) reported a nuclear localization signal (NLS) in IKK*α* (but not IKK*β*) that was essential for epidermal development. The reported data demonstrate abundant presence of nuclear IKK*α*, similar to pulmonary epithelium. Harhaj et al. found IKK*α* and NEMO (but not IKK*β*) in the nucleus at perinuclear “hot spots”, especially after HIV Tax expression, in modified Jurkat cells. Such “hot spots” could represent highly concentrated, oligomerized forms of IKK complexes, some of which were shown to overlap the Golgi complex; IKK*α* was detected in the nuclear fractions of resting human neutrophils (Ear et al. 2005).

The normal cellular distributions of IKK complexes are not defined. Kim inferred that CHC is linked to IKK*α* but the interactions in the NF‐kB interactome are not clear (Bouwmeester et al. 2004). CHC is a large cytoplasmic protein lacking a NLS, thus, it is typically excluded from the nucleus of interphase cells, although it can be perinuclear and even bind mitotic structures (Fotin et al. 2004; Sutherland et al. 2001). Our findings indicate that both IKK*α* and IKK*β* form complexes with CHC (Table 3, Figs 2–7).

Analysis of the cellular overlap of IKK*α*–IKK*β* complexes with either CHC or Hsp90 shows that a very small amount of the IKK*α*–IKK*β* colocalize with about half of the complexes made by IKK*β* with either protein. The number of voxels of IKK*α*–IKK*β* FRET complexes overlapping either IKK*β*–Hsp90 complexes (Fig. 2) or IKK*β*–CHC complexes (Fig. 3) is a small fraction of the total IKK*α*–IKK*β* FRET complexes (17% and 26%, respectively), but it constitutes a large portion of the IKK*β*–Hsp90 complexes (55%, Fig. 2) or IKK*β*–CHC complexes (50%, Fig. 3). This suggests that the complexes of IKK*β* with either Hsp90 or CHC may be precursors to the final IKK complex.

Analysis of the complexes of IKK*β* with either Hsp90 or CHC (Fig. 4) shows that about 74% of the voxels positive for IKK*β*–Hsp90 FRET overlap with 41% of voxels containing CHC–IKK*β* FRET and are restricted to perinuclear arcs. Figure 5 shows that there is only modest overlap (about 39 and 33%) of the individual complexes of IKK*α* with either Hsp90 or CHC, which are both typically perinuclear. In contrast, very few voxels (about 8%) showing NEMO–IKK*α* FRET overlap with 50% of IKK*α*–CHC FRET (Fig. 6) in the perinuclear compartment. Instead (Fig. 7), 22% of NEMO–IKK*β* FRET voxels are involved in overlap with 41% of IKK*β*–CHC FRET‐positive voxels in perinuclear and cytoplasmic compartments.

Although suggestive, the 99% confidence intervals for the overlapped fractions show a wide variation that indicates that the dependence of overlap on any one FRET pair may be treated better by regression analysis.

We therefore plotted numbers of voxels containing two FRET pairs against numbers of voxels containing each pair and found linear relationships with robust *P* values.



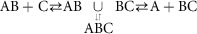



Either A, B, C may contain other subunits that are not visualized. We can assume that the voxels containing colocalized cFRETs may be related to “ternary” complexes (either preceding or following its dissociation). If the abundance of voxels containing colocalized cFRETs is linearly related to AB or BC complexes, the slope of the dependence suggests the direction of assembly.

In this first‐order model, we anticipated that, if the number of voxels containing colocalized cFRETs relate strongly to AB or BC complexes, the slopes of the dependence would indicate the overall direction of ternary assembly (allowing for disassembly rates). This regression exploits the variable amounts of individual FRET pairs in cells. The number of voxels containing colocalized cFRET pairs (denoted by Y) could indicate “ternary” complexes (either preceding or following dissociation). For this study, we focused only on complexes that can be confirmed by IP‐MS.

In most cases *r*^2^ values are above 0.76, showing excellent fit. *P* values for the calculated slopes are <1E‐6, except for two cases. Furthermore, the intercepts are mostly insignificant, supporting the simple linear dependence of overlap on FRET pair amounts.

Specifically, throughout the dynamic range, in unstimulated cells (i) the amount of IKK*α*–IKK*β* complexes has little effect on the overlap with IKK*β*–Hsp90 complexes suggesting that the two kinases together tend to not contain Hsp90. (ii) IKK*α*–IKK*β* clusters do not overlap with IKK*β*–CHC. (iii) the same is true for NEMO complexes with either IKK and CHC. (iv) In contrast, the number of complexes made by Hsp90 with either IKK influences overlap with the complexes made by the IKKs with CHC Thus, IKK–CHC complexes appear to lie downstream of the HSP90 complexes and precede the formation of IKK–NEMO complexes, but not as components of the IKK signalosome. The antibodies used in this study did not reveal whether the binary complexes involve CHC monomers, trimers, or even larger lattices. Similarly, we cannot discern the stoichiometry of the IKK components in the complexes.

The reported data demonstrate that labeling cellular proteins with antibodies after fixation offers complementary advantages to the more conventional tagging with GFP or other constructs. First, postfixation FRET in situ avoids problems due to the fractionation of fragile complexes. Moreover, it avoids potential problems associated with protein overexpression that can skew cellular contents, and to steric effects in fluorescent protein constructs (which can almost double the size of the protein) (Barken et al. 2005). Furthermore, indirect immunofluorescence with labeled secondary antibodies (spacers) addresses the challenge presented by the extended 47.5‐nm‐long CHC molecule.

These data agree with the recent findings of Hinz et al. (2007), Hayden and Ghosh (2008) that IKKs may exist as distinct complexes during maturation and that Hsp90 serves active catalytic IKK complexes at some point of the activation/inactivation cycle and/or stabilizes nascent complexes. The slopes (Table 3) indicate that the IKK*α*–IKK*β* complexes, although occurring in similar perinuclear and cytoplasmic areas, do not overlap systematically with IKK*β*–Hsp90 FRET. Rather, the amount of voxels where both FRETs are detected depends much more on the IKK*β*–HSP90 FRET. By those standards, the dependence on CHC is even less for IKK*α*–IKK*β* complex. Yet, we find a very strong dependence on IKK*β*–Hsp90 complexes that begins to overlap IKK*β*‐CHC conglomerates at perinuclear arcs. Moreover, both IKK–CHC complexes expand into IKK–NEMO‐rich areas, suggesting these CHC complexes somehow precede ternary IKK*α*–IKK*β*–NEMO assembly.

The proteomics and quantitative localization methodology outlined in this report could be utilized for studying other ternary interactomes of IKK*α* at natural levels or after perturbations such as knockdowns or receptor stimulation.

## Conflict of Interest

None declared.
